# Radiographic patterns of recurrence and pathologic correlation in malignant gliomas treated with bevacizumab

**DOI:** 10.2217/cns-2017-0025

**Published:** 2018-02-01

**Authors:** Alissa Thomas, Marc Rosenblum, Sasan Karimi, Lisa M DeAngelis, Antonio Omuro, Thomas J Kaley

**Affiliations:** 1 Department of Neurology, Memorial Sloan Kettering Cancer Center, 1275 York Avenue, New York, NY 10065, USA; 2 Department of Pathology, Memorial Sloan Kettering Cancer Center, 1275 York Avenue, New York, NY 10065, USA; 3 Department of Radiology, Memorial Sloan Kettering Cancer Center, 1275 York Avenue, New York, NY 10065, USA; 4 Department of Neurology, University of Vermont, 11 Colchester Avenue, Burlington, VT 05401, USA

**Keywords:** bevacizumab, glioblastoma, malignant glioma, necrosis, pathology

## Abstract

Interpretation of MRI abnormalities in patients with malignant gliomas (MG) treated with bevacizumab is challenging. Recent reports describe quantitative analyses of diffusion-weighted imaging abnormalities not available in standard clinical settings, to differentiate tumor recurrence from treatment necrosis. We retrospectively reviewed bevacizumab treated MG patients who underwent surgery or autopsy to correlate radiographic recurrence patterns with pathologic findings. 32 patients with MG (26 glioblastoma, three anaplastic astrocytoma and three anaplastic oligodendroglioma) were identified. Recurrence patterns: local enhancing (n = 23), distant enhancing (n = 1), nonenhancing (n = 7) and leptomeningeal (n = 1). Histology: tumor (n = 25), mixed tumor/necrosis (n = 5) and all necrosis (n = 2). On diffusion-weighted imaging, 5/32 had restricted diffusion (three mixed and two necrosis). Irrespective of radiographic recurrence pattern, tumor was found in 94% of cases. Restricted diffusion correlated with necrosis.

**Practice points**

- Patients with malignant gliomas treated with bevacizumab (BEV) are monitored with MRI scans, however the imaging changes may not always correlate with tumor response or progression.
- One such imaging parameter that is frequently studied is diffusion restriction.
- Some studies suggest that diffusion restriction is indicative of tumor progression that is ‘masked’ by BEV, while others suggest diffusion restriction indicates necrosis.
- Few studies actually include pathologic findings to provide a direct analysis of the diffusion restricted imaging abnormality.
- This study suggests that diffusion restriction correlates more with necrosis rather than tumor progression.
- Accurate interpretation of imaging in patients treated with BEV needs to be explored further to guide optimal use and management of BEV administration in patients with malignant gliomas.

Bevacizumab, a humanized recombinant monoclonal antibody directed against VEGF [1–3], induces rapid and potent radiographic responses in malignant gliomas (MG). MG secretes high levels of VEGF which promotes angiogenesis and vascular permeability to drive tumor progression [4–8]. Treatment with BEV is associated with improvements in progression-free survival in MG [9,10]. However, despite encouraging early responses, treatment with BEV does not translate to meaningful improvements in overall survival (OS) [11,12]. The disparity between response and survival highlights the limitations of contrast-enhanced MRI in predicting antitumor activity. By blocking VEGF, BEV induces change in the vasculature that suppresses the uptake of gadolinium and enhancement on MRI, regardless of any actual antitumor activity. To address this limitation many studies have adopted the Response Assessment in Neuro-Oncology (RANO) response criteria, which added significant increases in nonenhancing disease as a new criterion for disease progression [13].

Numerous studies have correlated MRI findings after treatment with BEV with survival outcomes [14–19]. In addition to standard MRI sequences of T1-weighted precontrast and postcontrast imaging, T2-weighted imaging and FLAIR, advanced imaging techniques including MR perfusion (MR-P), diffusion-weighted imaging (DWI), and fluorodeoxyglucose PET (FDG-PET) may aid the assessment of response to BEV. However, correlative studies give conflicting interpretations of MRI findings. For example, while some studies have suggested that DWI positivity with apparent diffusion coefficient correlate is predictive of recurrent tumor, others have reported the DWI lesions are most predictive of necrosis. DWI quantifies the Brownian movement of water molecules irrespective of directionality, assuming random and unrestricted diffusion [21]. Diffusion of water may be restricted by densely packed hypercellular tumor, or it may be inhibited by dead, necrotic tissue. Though it is well described that treatment with BEV is associated with DWI lesions [14,18,20,22–25], the significance of these lesions is controversial. Histopathologic data are limited [25], but much needed. Additionally, most of these studies utilized advanced radiologic metrics and quantitative imaging analyses which are not routine and thus not applicable to the standard MRI interpretation in the routine clinical setting.

In this retrospective analysis, we report radiographic and histopathologic outcomes of 32 patients with MG with clinical progression following treatment with BEV. We sought to identify the pathologic correlate of restricted diffusion in order to aid practitioners evaluating these patients in the clinic and faced with treatment decisions.

## Materials & methods

### Study design

This is an Institutional Review Board-approved retrospective single institution case series.

### Patient population

We retrospectively identified patients with MG, including glioblastoma, anaplastic astrocytoma and anaplastic oligodendroglioma, treated with BEV from an institutional database. We included only those patients who had tumor tissue available after BEV treatment, either from re-resection or autopsy. We reviewed the charts for demographic information including age, gender, Karnofsky performance status and survival data. We also collected treatment details, including BEV dose and duration, prior chemotherapy or radiation therapy, concurrent therapy and treatment following BEV.

### Radiology review

MRI scans were reviewed and classified as enhancing or nonenhancing at recurrence. We also reviewed the MRIs for the presence or absence of diffusion restriction by standard qualitative radiology criteria at the time of radiographic recurrence. In the subset of patients who had advanced imaging at the time of progression, we also examined the MR-P and/or FDG-PET scan to determine if the tumor progression was hyper- or hypo-perfused and hyper- or hypo-metabolic, respectively. MR-P was performed using dynamic susceptibility contrast (DSC) MRI.

### Pathology review

Pathology specimens were taken after BEV treatment, either when patients underwent re-resection for presumed disease progression or at autopsy for BEV failure. Pathology specimens were examined by the study pathologists and classified as tumor, necrosis, or mixed tumor and necrosis.

### Statistics

A two-tailed Fisher exact test was used to compare the imaging characteristics and correlation with tumor/mixed tumor versus necrosis. The significance level was set at an α of 0.05.

## Results

### Patient characteristics

We identified 32 patients with MG treated with BEV who underwent surgical resection or autopsy following presumed tumor progression (radiographic recurrence/progression) while on BEV (Table 1). The median age at the time of tumor diagnosis was 56 years (range: 18–81). There were 25 men (78%) and seven women. The median Karnofsky performance status was 90% (range: 70–100). 26 patients (81%) were diagnosed with glioblastoma, three with anaplastic astrocytoma (9%) and three with anaplastic oligodendroglioma (9%).

**Table 1.** **Patient characteristics.**

**Patients**	**n = 32**
Tumor histology: – Glioblastoma – Anaplastic astrocytoma – Anaplastic oligodendroglioma	n = 26 (81%) n = 3 (9%) n = 3 (9)

Gender: – Men – Women	n = 25 (78%) n = 7 (22%)

Median age (years)	56 (range 18–81)

Median KPS	90 (range 70–100)

BEV treatment: – At diagnosis – At recurrence	n = 22 (68%) n = 10 (31%)

Post-BEV pathology: – Resection – Autopsy	n = 26 (81%) n = 6 (19%)

BEV: Bevacizumab; KPS: Karnofsky performance status.

### Treatment characteristics

Ten patients (31%) were treated with BEV at initial diagnosis, concurrently with radiation therapy and chemotherapy. 22 patients (69%) received BEV at recurrence. All of the patients treated with BEV at recurrence had previously received radiation therapy and temozolomide and eight had received a second course of radiation therapy. Among the 22 patients treated at recurrence, 13 received BEV alone and nine received bevacizumab in combination with chemotherapy, including three with temozolomide, five with irinotecan and one with lomustine. 31 out of 32 patients (97%) received BEV at a dose of 10 mg/kg every 2 weeks and one patient was dosed at 15 mg/kg every 3 weeks. The median number of BEV doses was 12 with a range of 3–70 doses. All of the patients died during the follow-up period. The median OS from the start of BEV for the newly diagnosed cohort was 15.3 months (range 7.2–35.4 months) and 13.8 months for the recurrent cohort (range 5.1–50.4 months).

### Radiographic patterns & pathologic correlates

All patients had an MRI of the brain with and without contrast and with DWI at the time of suspected disease progression or prior to death. 12 patients had MR-P and four had FDG-PET scans (Table 2).

**Table 2.** **Imaging characteristics and pathologic correlates.**

**Imaging characteristics (n)**	**Pathology (n)**		

	***Tumor (25)***	***Mixed tumor/necrosis (5)***	***Necrosis (2)***
***Enhancement pattern (n = 32)***			

Enhancing (25)	19	4	2

Nonenhancing (7)	6	1	0

***DWI characteristics (n = 32)***			

Restricted diffusion (5)	0	3	2

No restricted diffusion (27)	25	2	2

***MR-P characteristics (n = 12)***			

DSC-MRI hyperperfusion (8)	7	1	0

DSC-MRI Hypoperfusion (4)	0	2	2

***FDG-PET characteristics (n = 4)***			

FDG-PET Hypermetabolic (1)	0	1	0

FDG-PET Hypometabolic (3)	2	1	0

DWI: Diffusion-weighted imaging: FDG-PET: Fluorodeoxyglucose PET; MR-P: MR perfusion.

On MRI, 23 patients had local enhancement, one distant enhancing recurrence, seven nonenhancing recurrence and one diffuse leptomeningeal recurrence. Of the 32 patients, 30 (94%) had pathology specimens with tumor (25 pure tumor, five were tumor and necrosis) and two demonstrated necrotic tissue only. In the seven patients with nonenhancing disease, all had tumor at pathology, one of which was mixed tumor and necrosis. All eight patients with hyperperfusion on DSC-MRI had tumor confirmed by pathology (seven pure tumor, one mixed tumor and necrosis). Among the four patients with hypoperfusion on DSC-MRI, two had mixed tumor and necrosis and two had all necrotic specimens. Hyperperfusion was associated with tumor pathology and approached statistical significance (p = 0.09).

**Figure 1.**
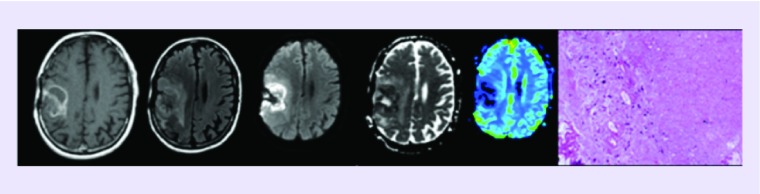
**Patient demonstrating (from left to right) axial T1-weighted post-contrast MRI, FLAIR, diffusion-weighted imaging, apparent diffusion coefficient and dynamic susceptibility contrast perfusion preoperatively, followed by pathologic specimen demonstrating a few degenerating glioma cells in a background of extensive necrosis.**

All patients had MRI that included DWI and apparent diffusion coefficient at the time of progression. There were five patients with restricted diffusion on MRI, three of whom had mixed tumor and necrosis and two with necrosis only. All the 27 patients without restricted diffusion had tumor (25 pure tumor, two mixed tumor and necrosis) on pathology. Diffusion restriction was associated with necrosis (p = 0.02; Figure 1). Of note, the two patients with only necrosis lived 9 months and 18 months after the procedure demonstrating necrosis supporting the diagnosis of necrosis.

## Discussion

BEV is a potent inhibitor of VEGF, the primary driver of angiogenesis in glioblastoma, and it plays an important role in the treatment of recurrent MG. BEV often improves neurologic symptoms related to cerebral edema and enhancing tumor progression [26]. A large proportion of patients with MG have a radiographic and/or clinical response to BEV, but the patterns of failure after BEV treatment are less well understood. In our cohort of 32 patients with clinical progression on BEV, 94% were found to have tumor on post-BEV pathology specimens. The two patients (6%) who had necrosis exclusively on histology had MRI findings of enhancing tumor on T1-weighted postcontrast imaging and diffusion restriction on DWI.

The significance of restricted diffusion in glioblastoma is controversial, in the presence or absence of BEV. In one study, 67 out of 208 (32%) patients with glioblastoma, regardless of treatment, developed DWI lesions [20]. Nearly half of these patients went on to develop enhancement at the site of the DWI lesion, a median of 3 months after the appearance of the restricted diffusion. DWI predicted later enhancement regardless of treatment with BEV. All patients with DWI lesions either worsened clinically or died from the disease. Lesions with restricted diffusion may correlate with increased tumor cellularity, glioma grade, cellular proliferation and ischemia [20,27–29]. In untreated gliomas, hypercellularity is likely the primary cause of restricted diffusion, and it correlates worse prognosis [20,30–34]. Following treatment, cell death, necrosis, edema, gliosis, hemorrhage and mineralization, all affect the Brownian movement of water and therefore may contribute to restricted diffusion.

In BEV treated tumors, hypoxia due to insufficient vascular proliferation may contribute to formation of DWI positive lesions [20,35]. Restricted diffusion is frequently reported after treatment with BEV. One study reported the appearance of DWI lesions in 12 patients treated with radiation therapy followed by BEV [22]. DWI lesions appeared 3–21 months after radiation and 1–6 months after BEV, and increased in size over time. In another study of 18 patients with recurrent MG treated with BEV, 13 developed DWI lesions within the previously enhancing tumor, a median of 4 weeks after initiation of BEV [23].

The significance of restricted diffusion following treatment with BEV is controversial. While studies of clinical correlation suggest that DWI lesions are associated with tumor progression, pathology studies have not supported this. One study reported 21 patients with BEV-treated recurrent glioblastoma who developed DWI lesions that were also T1 precontrast hyperintense [15]. These patients had a much worse survival than their counterparts who did not have DWI and T1-bright lesions (OS: 6.6 vs 13 months); one possible interpretation of these data is that the restricted diffusion in this study was due to hypercellular tumor growth which could explain the worse prognosis. However, our study and other case reports of the pathology of DWI lesions in BEV-treated MG patients have all demonstrated necrosis in the absence of viable tumor [23,25]. We note that our patients with necrosis had enhancement at the DWI-positive site, and it is possible that nonenhancing recurrent tumor may be DWI-positive related to hypercellularity.

Aside from DWI, MR-P may be helpful in understanding patterns of recurrence after BEV treatment. In our study we used DSC-MRI to identify lesions with increased relative cerebral blood volume. Studies have shown that patients with hyper-perfused tumor prior to treatment with BEV have longer survival following BEV treatment [16,36]. Following treatment with BEV, increased perfusion as measured by high relative cerebral blood volume values on DSC-MRI correlated with poor OS and progression-free survival. In our study, all eight patients with hyperperfusion on DSC-MRI had tumor on their specimens.

Only two of our 32 patients (6%) presented with distant or diffuse (leptomeingeal) disease at the time of progression following BEV treatment. Hypoxia is known to stimulate tumor growth and invasiveness [25,39]. Some researchers have hypothesized that BEV may increase the invasiveness of tumor cells by increasing tumor hypoxia [18,37,38]. However, the majority of MG treated with BEV recur locally. One study demonstrated that 82% of MG treated with BEV recurred at the primary site of disease. Others have demonstrated similar rates of distant or disseminated recurrence in glioblastoma treated with or without BEV [40].

Our study is limited by its small sample size and retrospective design. The small size, retrospective nature and limited number of patients who had MR-P and PET imaging precludes us from making predictions about histology based on the radiolographic findings. There is inherent bias toward finding viable tumor because 19% of our samples were from autopsies. However, to our knowledge this is the largest series of pathology specimens reported after BEV treatment.

## Conclusion

Recurrent MG may present with enhancing or nonenhancing lesions on standard MRI following BEV treatment. Advanced imaging techniques including DWI, MR-P and FDG-PET may be helpful in differentiating recurrent tumor from BEV-induced necrosis, but more histopathological data are needed to help us to better interpret MRI findings. When practitioners encounter DWI positive abnormalities in BEV-treated MG patients, we do not yet know how to accurately assess these patients on standard clinical imaging. Even though BEV is sometimes considered a treatment for necrosis, our study demonstrates that it is still possible to develop necrosis during treatment with BEV and this may be evident on diffusion-weighted sequences.

## Future perspective

Managing patients with MG is challenging and BEV is a particularly challenging therapy because it may change various imaging parameters as a function of the drug itself without necessarily being a function of its effect on the tumor. Better imaging modalities are needed to appropriately assess patients receiving BEV as well as other newer anticancer therapies in the clinic. This will allow proper assessment of the success or failure of these therapies in this disease. We tried to include MR-P imaging which may play a role in the future. We also tried to include FDG-PET where it was available. Although alternative PET tracers may be more effective at distinguishing necrosis from tumor and many of these trials are ongoing, they are not yet available routinely in clinical practice.
